# An Insight Into the Effect of Organic Amendments on the Transpiration Efficiency of Wheat Plant in a Sodic Duplex Soil

**DOI:** 10.3389/fpls.2021.722000

**Published:** 2021-10-20

**Authors:** Xiaojuan Wang, Peter Sale, Ashley Franks, Jian Jin, Christian Krohn, Roger Armstrong, Caixian Tang

**Affiliations:** ^1^Department of Animal, Plant and Soil Sciences, AgriBio-Center for the AgriBiosciences, La Trobe University, Bundoora, VIC, Australia; ^2^Department of Physiology, Anatomy and Microbiology, La Trobe University, Bundoora, VIC, Australia; ^3^Center for Future Landscapes, La Trobe University, Bundoora, VIC, Australia; ^4^Department of Jobs, Precincts & Regions, Grains Innovation Center, Horsham, VIC, Australia

**Keywords:** *Bacillus*, organic amendments, plant beneficial bacteria, stomatal conductance, subsoil constraints, transpiration efficiency

## Abstract

Transpiration efficiency, the shoot biomass produced per unit of transpired water, is generally considered to be a constant property for a given crop in a given environment. To determine whether deep-banded organic amendments affect the transpiration efficiency (TE) of wheat plants and to provide a possible explanation for any changes in the TE, two-column experiments were carried out under controlled environment conditions. A Sodosol soil with physically constrained subsoils and a well-structured Vertosol were subjected to treatments including a control, fertilizer nutrients alone, and fertilizer-enriched organic amendments. The addition of fertilizer-enriched organic amendments in Sodosol consistently increased the canopy TE compared to the control and inorganic fertilizer treatments. The instantaneous TE, at the leaf level, was also increased by the organic-based amendments due to greater reductions in stomatal conductance and transpiration rates during periods of moderate water-deficit stress and the subsequent recovery from this stress. Shoot nitrogen (N) status could not explain the increases in TE following the addition of organic amendments relative to inorganic amendments. The increases in canopy TE were directly associated with increases in the absolute abundance of indigenous *Bacillus* (*R*^2^ = 0.92, *p* <0), a well-known genus comprising many strains of plant beneficial rhizobacteria, in subsoil below the amendment band. In contrast, there were no differences in the canopy TE and instantaneous leaf TE between the organic and fertilizer amendments in the Vertosol with a well-structured subsoil. The positive effect of organic amendments on TE in the Sodosol should be attributed to their direct or indirect effect on improving the physical structure or biological properties of the subsoil.

## Introduction

The ultimate price paid by land plants for photosynthesis is the unavoidable loss of water *via* transpiration. Stomata in the leaves surface open to let atmospheric CO_2_ diffuse into leaf cavities, but at the same time enable water vapor to diffuse out to the atmosphere (von Caemmerer and Baker, 2007). Consequently, the grain yield of field crops links directly to the amount of water transpired by the leaf canopies in water-limited environments.

The water-limited yield (Y) of rain-fed crops has been defined by Passioura (1996) using the relationship

Y_(water−limited)_ = T _(transpiration)_ × TE _(transpirationefficiency)_ × HI _(harvestindex)_

where T is total transpiration, TE is the transpiration efficiency (total shoot biomass/total transpiration), and HI is the harvest index (total grain yield/total shoot biomass). A key objective for rain-fed crops is to maximize the portion of soil water that is transpired by crops relative to water loss due to evaporation. Increasing transpiration generally increases the harvest index (Stewart and Lal, 2018) and crop yield, particularly for water transpired after flowering. Similarly, an increase in TE would also increase crop yields in a water-limited environment. However, the TE of a crop depends on relative humidity and the nature of the crop and hence is relatively constant for a given crop genotype in a given environment (Ehlers and Goss, 2016).

Transpiration efficiency at the canopy level is an integral of the instantaneous or intrinsic TE at the leaf level. The underlying mechanism for the intrinsic TE in wheat leaves has been expressed by Condon et al. (2002) as

TE_intrinsic_ = 0.6 Ca × (1-Ci/Ca)/(Wi-Wa)

where Ci/Ca is the ratio of the CO_2_ concentration in the sub-stomatal cavity, to that in the atmosphere. The Wi-Wa is the vapor pressure deficit, which is the difference in vapor pressure between the stomatal cavity and the ambient environment. These terms have been extensively investigated in crop breeding programs with the aim of increasing intrinsic TE. For example, an increase in TE can be achieved by reducing Ci *via* the genetic manipulation of stomatal conductance (the rate of gas exchange through the stomata) or by increasing leaf photosynthetic activity (Faralli et al., 2019). Other studies found that species capable of restricting transpiration by partially closing their stomata under high vapor pressure deficits have shown a lower transpiration rate and therefore a higher TE (Rebetzke et al., 2002; Zhang et al., 2019). Similar to the leaves, response to air dryness, plant roots are also able to sense soil dryness and, in response, synthesize hormones such as abscisic acid (ABA) to induce stomatal closure and reduce the transpiration rate (Kondo et al., 2004; Cabrera-Bosquet et al., 2007; Sinclair, 2012). Thus, the ability of plants to control stomatal opening and closing, in response to environmental conditions, will allow for the conservation and efficient use of water.

More than 70 years of research following the detailed study by de Wit (1958) has shown limited evidence of any direct effect of soil management on TE. Increases in the water use efficiency in the field following the addition of inorganic fertilizer are mainly attributed to reductions in soil water evaporation relative to plant transpiration, rather than to any increase in intrinsic TE (Passioura and Angus, 2010). However, there have been several published reports of increased TE with the addition of organic manures to the topsoil (Wang et al., 2017) or to the subsoil (Espinosa et al., 2011). Also, a series of unpublished reports in our laboratory have consistently shown how the banding of poultry litter in poorly structured sub-surface layers of Sodosol has increased the TE of the wheat plant, although the basis for this effect on TE is not understood. Ample evidence showed that the direct inoculation of plant seeds or roots with beneficial bacteria such as *Bacillus subtilis* or *Bacillus licheniformis* FMCH001 could enhance plant photosynthetic capacity (Li et al., 2016; Barnawal et al., 2017) or reduce water stress *via* partial stomatal closure (de Lima et al., 2019; Akhtar et al., 2020). Possibly, organic amendments enable plants to use water more efficiently *via* their direct effect on soil physical and/or biological properties.

Here, we report on two experiments where wheat plants were grown in field-simulated Sodosol soil columns with dense clay subsoils. The objective of the first experiment was to confirm whether deep-banded, nutrient-rich organic amendments can increase TE at the canopy and at the leaf level. The second experiment focused on stomatal function in the youngest fully expanded leaves of the wheat plant and how this is affected by organic amendments during the drying and rewetting of the soil. In addition, the composition of the bacterial community in the subsoil, associated with the amendments, was measured in order to provide a possible explanation for any changes in the TE. A Vertosol soil with a well-structured subsoil was included as a contrasting soil type to the Sodosol, to indicate whether subsoil constraints might interact with amendments in affecting TE.

## Materials and Methods

### Soils

The soil used in both experiments was collected from the 0–10 cm (topsoil) and the 20–50 cm deep subsoil layers from a cropping paddock (37.88°S, 144.23°E) on Yaloak Estate, a farm near Fiskville in south-west Victoria. The soil is classified as a Sodosol (Isbell, 2016) or Solonetz (WRB and IWG, 2015). The topsoil is sandy loam while the subsoil is dense and sodic with an exchangeable sodium percentage (ESP) of 21% and clay content of 53.7%. A Vertosol (Isbell, 2016) or Vertisol (WRB and IWG, 2015) with well-structured clay topsoil and subsoil were also collected from the 0–10 to 20–50 cm layers from a farmer's field in northwestern Victoria as a comparison in Experiment 2. Selected properties of the topsoil and subsoil are presented in Supplementary Table 1. All soils were air-dried and broken down to pass through a 2-mm sieve.

### Experimental Design and Treatments

Experiment 1 was laid out as a randomized complete block design. There were five amendments that included a control (no amendment), fertilizer nutrients (NPKS), wheat straw + fertilizer nutrients (straw/NPKS), poultry litter (PL), and poultry litter + macracote (PL/mac). Treatments were replicated three times, and soil columns were destructively harvested at stem elongation and maturity. The NPKS treatment involved mixing 10 g of dry soil, with 0.94 g of diammonium phosphate (DAP), 0.72 g urea, and 1.18 g K_2_SO_4_ column^−1^ which were equivalent to 300 kg N, 125 kg P, 300 kg K, and 123 kg S ha^−1^ on a surface-area basis, respectively. To rule out the effect of differences in nutrient supply, especially N, additional inorganic nutrients were added together with organic amendments to match the total available nitrogen (N) in the NPKS treatment. Hence, the straw/NPKS treatment involved mixing fertilizer solutions thoroughly with the chopped wheat straw pieces (<1 cm) in the same form and rates as the NPKS treatment. The PL/mac treatment involved the same amount of poultry litter as the PL treatment, to which Macracote Orange, a controlled-release inorganic fertilizer (Langley Fertilizers, Australia), was added at 7.5 g column^−1^ to provide plant available N equivalent to that of NPKS treatment. The poultry litter and wheat straw amendments were added on an equivalent surface area basis of 20 t ha^−1^ (35.3 g column^−1^).

The aim of Experiment 2 was to confirm the findings from Experiment 1 and to provide insight into potential mechanisms. This experiment was set up using the same amendment treatments as Experiment 1 except that urea, rather than macracote, was added together with PL. The results from Experiment 1 indicated that N released from macracote was much higher than that calculated based on its release rates over 120 days at 20°C (Langley Fertilizers). Therefore, 0.738 g urea was mixed with 35.3 g PL (PL/N) to provide an additional 339.3 mg N per column in Experiment 2, assuming that N released from PL at wheat maturity was close to 15% of its total N (Wang et al., 2020). For the Vertosol, three treatments included a control, and the NPKS and PL/N treatments and were replicated three times.

### Column Set-Up

The experimental unit was a 40-cm high PVC column with an internal diameter of 15 cm along with a modification in Experiment 1 which used a dual-column system to minimize root growth down the sides of PVC columns (Wang et al., 2020). The soil columns were constructed as follows: 24 cm of topsoil (in the 10 cm in diameter PVC tube in Experiment 1) or 8 cm of topsoil (in the 15 cm in diameter PVC pipe in Experiment 2) overlaid on 32 cm of subsoil. Basal nutrients were added to the topsoil at the following rates (mg kg^−1^): CO(NH_2_)_2_, 86; KH_2_PO_4_, 180; K_2_SO_4_, 180; CaCl_2_.2H_2_O, 180; MgSO_4_.7H_2_O, 50; MnSO_4_.H_2_O, 15; ZnSO_4_.7H_2_O, 9; CuSO_4_.5H_2_O, 6; Na_2_MoO_4_.2H_2_O, 0.4; and FeEDTA, 5.5. The subsoil was packed layer by layer with consistent tapping and watering to reach a final bulk density of 1.3 and 1.4 g cm^−3^ in Experiments 1 and 2, respectively. The higher bulk density in Experiment 2 aimed to maximize differences in physical structure between Sodosol and Vertosol subsoil and to mimic what occurred in the field. The amendments were added into the subsoil, 6 cm below the bottom of the topsoil. A plastic, perforated watering tube, formed into a circular 10 cm diameter ring, was placed at the center of the column some 6 cm below the amendments for use in supplying water to the subsoil. The surfaces of the topsoil were covered with a 3-cm layer of plastic beads to prevent surface evaporation. Three spare soil columns, set up in a similar manner, were used without plants to determine non-transpiration water losses from the columns. All soil columns were wet to 80% of field capacity and pre-incubated for 1 month at 25°C.

### Growing Conditions

Both experiments were conducted in a controlled-environment room with conditions set to a 14-h day at 25°C, a 10-h night at 18°C, and a light intensity of 450 μmol m^−2^ s^−1^. Eight pre-germinated seeds of wheat (*Triticum aestivum* cv. Yipti) were sown in each column. After emergence, the plants were thinned to three and four uniform seedlings per column in Experiment 1 and Experiment 2, respectively. All columns were weighed and watered, either from the topsoil initially or increasingly from the subsoil watering tube, every 1 or 2 days with deionized water, to maintain 80% field capacity.

### Daily Water Use and Leaf Gas Exchange Measurements

The daily water use was recorded by weighing all columns every 1 or 2 days before each watering event. The tiller number was counted and the Soil Plant Analysis Development (SPAD) readings that measure a chlorophyll index were carried out on the youngest fully expanded leaves weekly. In Experiment 2, the CO_2_ and water vapor exchange rates were measured using the LCi Portable Photosynthesis System (ADC Bioscientific, Hoddesdon, UK) on the youngest fully expanded leaves of the main stem. The measurements occurred 2 h after each watering event on leaves from three plants per column for selected treatments during the tillering period (days 24–43) and then during the stem elongation to the ear emergence phase (days 49–60). On days 31, 39, and 42, the gas exchange measurements were also taken before each watering event when the averaged soil water content in the soil column was <24%. These plants were considered to be under moderate water stress due to mild wilting of the lower leaves after the water had been withheld for more than 28 h. Where soil moisture content was maintained above 26%, the plants were considered to be well-watered and were not under any water stress at any time (e.g., at the early tillering stage).

### Shoot, Root, and Soil Sampling

At harvest, shoots in each column were removed at the soil surface, then washed and oven-dried at 70°C for 48 h before weighing. At maturity, threshed grains were also oven-dried and weighed. All columns were sectioned into the topsoil layer, the amendment layer containing the amendment bands (8- cm), and the subsoil layer that was 30–32 cm deep, to collect roots in each layer for root length and dry weight measurements. For bacterial measurements in Experiment 2, soil samples were collected 1 cm below the amendments (2–3 cm thick) and 1 cm away from the wall of the PVC column. All soil samples were broken up into small pieces and mixed gently before collecting 50 g subsamples and then storing them immediately at −80°C for genomic DNA extraction. After soil sampling, roots were recovered by soaking the remaining soil from each layer in water for several hours and then gently washing the soil from the roots over a 1-mm sieve. The washed roots were then cut into 3–4 cm segments, evenly mixed, and subsampled for root scanning using an EPSON EU-35 scanner (Seiko Epson Corp, Suwa, Japan) and the WinRHIZO STD 1,600+image analysis system (Regent Instruments, Quebec City, Canada) to measure total root length. After scanning, the roots were oven-dried at 70°C and weighed. Shoots and grains were ground, ball-milled, and analyzed for N using a CHNS Analyzer (PerkinElmer EA2400, Shelton, CT, USA).

### DNA Extraction, Quantitative PCR, and Sequencing for Bacteria

Soil microbial DNA was extracted from 0.25 g soil samples using the Mobio PowerSoil DNA Isolation Kit as per the instructions of the manufacturer. DNA concentration and purity were determined using an Implen P330 NanoPhotometer (Implen GmbH, Munich, Germany) and Qubit 3.0 Fluorometer platform [ThermoFisher Scientific; Research Facility (Melbourne, Australia) on an Illumina MiSeq (Illumina Inc., CA, USA)].

Quantitative PCR (qPCR) was used to quantify the total bacterial copy number on a CFX Connect Real-Time PCR Detection System (Bio-Rad, Hercules, CA, USA). The primer sets of 1114f (5′CGGCAACGAGCGCAACCC) with 1275r (5′CCATTGTAGCACGTGTGTAGC) were used to target the bacterial 16S rRNA gene (Denman and McSweeney, 2006). To create a bacteria standard curve, DNA extracted from *E. coli* DH5 alpha were amplified with 1114f-1275r, run on a 1% (w/v) agarose gel, excised, pooled, purified using a Bioline Isolate II PCR and gel clean-up kit (Bioline International, Toronto, Canada), and then followed by a serial dilution of the purified amplicon from 1^−1^ to 10^−8^. The bacterial 16S rRNA assays were carried out in 5 μl reactions containing 0.5 μl of each primer (10 μM), 2.5 μl of SsoAdvanced Universal SYBR® Green Supermix (Bio-Rad), and 0.5 μl sterile DNA-free water and 5 ng of template DNA. Thermal cycling conditions were 20 s at 95°C followed by 40 cycles of 95°C for 3 s and 61.5°C for 30 s. Reactions were followed by a melting curve increasing 1°C every 30 s from 60°C to 99°C. Each 96-well plate contains triplicate standard curves and triplicate negative (no-temple controls). For each sample, the qPCR measurement was duplicated, and the average efficiency was 91.6 % and *R*^2^ was 0.994.

The V4 hypervariable region of the bacterial 16S rRNA genes was amplified with primers 515F (5′-GTGYCAGCMGCCGCGGTAA) and 806R (5′GGACTACNVGGGTWTCTAAT), and sequenced on an Illumina MiSeq platform (Caporaso et al., 2012). The paired raw sequences were demultiplexed, dereplicated, and quality filtered using default settings of the dada 2 plugin within the framework of Quantitative Insights into Microbial Ecology 2 (QIIME 2, v2020.2) with 4,254,078 (74%) quality sequences retained and a median frequency of 60,583 sequences per sample (Callahan et al., 2016). Furthermore, 21,407 unique amplicon sequence variants (ASVs) were identified, and their taxonomy was classified using pre-trained silva classifiers (v132) at 99% similarity. The absolute abundance of each bacterial genus was calculated by multiplying total bacterial copy numbers from qPCR and the corresponding relative abundance determined from high-throughput sequencing (Lou et al., 2018).

### Statistical Analysis

A one-way ANOVA was conducted to assess the effects of amendments at each growth stage and for each soil. Log transformations of data were used when data failed to meet the assumptions of normality for the analysis of variance, including the bacterial data. Significant differences between means were identified using Duncan's multiple range test. All statistical analyses were performed using Genstat (11th version; VSNI Hertfordshire, UK). The linear relationships between the absolute *Bacillus* abundance and tiller number at day 35 or root length density or plant TE were determined by linear regression using Excel for Windows 10.

## Results

### Growth and Water Relations of Wheat Plants

Deep-banded amendments had a marked effect on the growth, N status, and water relations of wheat plants in both experiments (Tables 1, 2). In Experiment 1, the most productive amendments were the fertilizer-enriched organic amendments: PL/mac and straw/NPKS with the latter being notable in its effect on increasing root biomass. At maturity, the PL/mac amendment produced the largest shoot biomass (96.4 g column^−1^) and ear numbers followed by the straw/NPKS (75.5 g column^−1^), NPKS (68.0 g column^−1^), and PL amendments (60.2 g column^−1^) (Table 1). When compared to the control, the grain yield increased by 103, 50, and 41% in the Sodosol amended with PL/mac, straw/NPKS, and NPK, respectively. The PL produced similar shoot biomass to NPKS at stem elongation, despite substantially lower shoot N concentrations. Symptoms of N-deficiency were apparent in the PL-amended soil at early stem elongation.

**Table 1:** Effect of amendments added in the Sodosol subsoil on the shoot and root biomass, grain yield, N concentration, total water-use, and transpiration efficiency (TE) at stem elongation (7 weeks) and at maturity (16 weeks) in Experiment 1.

**Treatments**	**Tiller/ear No**	**Biomass**		**Shoot N conc**.	**Grain**		**Total water use**	**TE**	
	**(column^**−1**^)**	**Shoot**	**Root**		**Yield**	**N conc**.		**canopy**	**Grain**
		**(g column** ^**−1**^ **)**		**(mg g^**−1**^)**	**(g column^**−1**^)**	**(mg g^**−1**^)**	**(l column^**−1**^)**	**(g l** ^**−1**^ **)**	
**Stem elongation**									
Control	24.6 a	9.9 a	2.9 a	30.0 ab			3.6 a	2.73 a	
NPKS	46.2 b	15.4 b	3.2 a	42.4 c			4.8 c	3.19 b	
NPKS/straw	51.0 c	19.0 c	4.1 c	31.8 b			5.1 c	3.72 c	
Poultry litter	44.0 b	15.2 b	3.8 b	28.4 a			4.3 b	3.55 c	
Poultry litter/mac	51.6 c	20.0 c	3.9 b	44.1 c			4.8 c	4.17 d	
**Maturity**									
Control	16.7 a	43.7 a	2.7 a	2.6 a	19.2 a	17.7 a	14.9 a	2.94 a	1.29 b
NPKS	24.3 c	68.0 c	3.5 b	3.7 b	27.1 bc	21.6 b	24.3 c	2.80 a	1.12 a
NPKS/straw	27.0 d	75.5 d	4.8 c	2.8 a	28.8 c	18.4 a	23.8 c	3.17 b	1.21 b
Poultry litter	20.7 b	60.2 b	3.7 b	2.6 a	24.9 b	18.4 a	19.7 b	3.06 a	1.26 b
Poultry litter/mac	32.3 e	96.4 e	4.5 c	3.8 b	38.9 d	21.2 b	30.2 d	3.19 b	1.29 b

*Different letters indicate significant differences between treatments (p < 0.05) at each growth stage using Duncan's multiple range test*.

**Table 2:** Effect of amendment treatments on the shoot and root growth, nitrogen (N) concentration, total water use, and TE in Sodosol and Vertosol soils over 62 days in Experiment 2.

	**Tiller No**		**Ear**		**Biomass**		**Shoot N conc**.	**Total water use**	**TE**
	**Day 20**	**Day 35**	**No**	**Weight**	**Shoot**	**Root**			
	**(column** ^**−1**^ **)**		**(column^**−1**^)**	**(g column^**−1**^)**	**(g column** ^**−1**^ **)**		**(mg g^**−1**^)**	**(l column^**−1**^)**	**(g l^**−1**^)**
**Sodosol**									
Control	17.0 a	24.3 a	11.7 a	4.4 a	18.8 a	3.1 a	12.0 a	6.7 a	2.82 a
NPKS	21.0 a	44.3 b	20.3 bc	8.3 b	40.2 b	4.7 b	14.1 b	12.5 c	3.21 b
NPKS/straw	27.0 b	50.5 c	22.7 c	10.9 c	46.7 c	6.2 d	11.0 a	12.6 c	3.72 c
Poultry litter	30.5 b	47.0 bc	19.7 b	8.4 b	38.8 b	5.4 c	11.2 a	11.1 b	3.50 c
Poultry litter/N	30.5 b	58.7 d	23.7 c	10.4 c	48.5 c	5.6 cd	13.0 b	13.3 c	3.65 c
**Vertosol**									
Control	30.7	35.0 a	12.7 a	5.2 a	30.6 a	4.1 a	8.4 a	8.7 a	3.53 a
NPKS	30.0	64.7 b	22.7 b	11.6 b	65.1 b	9.3 b	9.8 b	17.3 b	3.76 ab
Poultry litter/N	29.0	67.3 b	25.3 b	11.7 b	70.7 b	9.6 b	9.4 b	17.6 b	4.08 b

*Different letters indicate significant differences (p ≤ 0.05) between treatments for each soil using Duncan's multiple range test*.

At stem elongation, there was a large increase in canopy TE relative to the control with the PL/mac amendment (52.7%) and straw/NPKS (36.3%). The basis for this, in most instances, was that PL/mac and straw/NPKS produced higher shoot biomass and grain yields compared with NPKS but transpired similar amounts of water (Table 1). The PL amendment showed the same shoot biomass to NPKS at stem elongation yet transpired considerably less water and hence, had a higher TE. At maturity, the canopy TE was 8.5% higher (*p* < 0.01) with the NPKS/straw and PL/mac amendments compared to other treatments, and there were no differences in canopy TE between the control, NPKS, and PL amendments. The grain TE was less affected by the amendments, except for NPKS which decreased the grain TE by 13% relative to the control.

Similar results occurred with the same Sodosol soil in Experiment 2 when plants were harvested at the ear emergence stage (Table 2). The fertilizer-enriched organic amendments PL/N and straw/NPKS again were the most productive amendments in terms of tiller numbers, ear density and ear mass, and shoot and root biomass. In addition, canopy TE was significantly higher with the PL, PL/N, and straw/NPKS amendments compared to the NPKS amendment. Again, the shoot biomass was larger, and similar quantities of water were transpired with PL/N and straw/NPKS compared to NPKS. The PL amendment produced the same shoot biomass as NPKS, but less water was transpired (Table 2). Similar to Experiment 1, the highest shoot N concentrations were detected for the NPKS and PL/N. The shoot N concentration was low for PL and straw/NPKS, which did not differ from that of control.

The pattern of wheat responses to amendments differed between Vertosol and Sodosol (Table 2). In contrast to the Sodosol, there were no differences in plant growth, water use, or canopy TE between the PL/N and NPKS amendments in the Vertosol (Tables 1, 2). Moreover, wheat plants grew much better in the Vertosol compared with the Sodosol as there were more tillers at day 35, higher ear mass, shoot and root biomass, and greater total water use.

**Figure 1:**
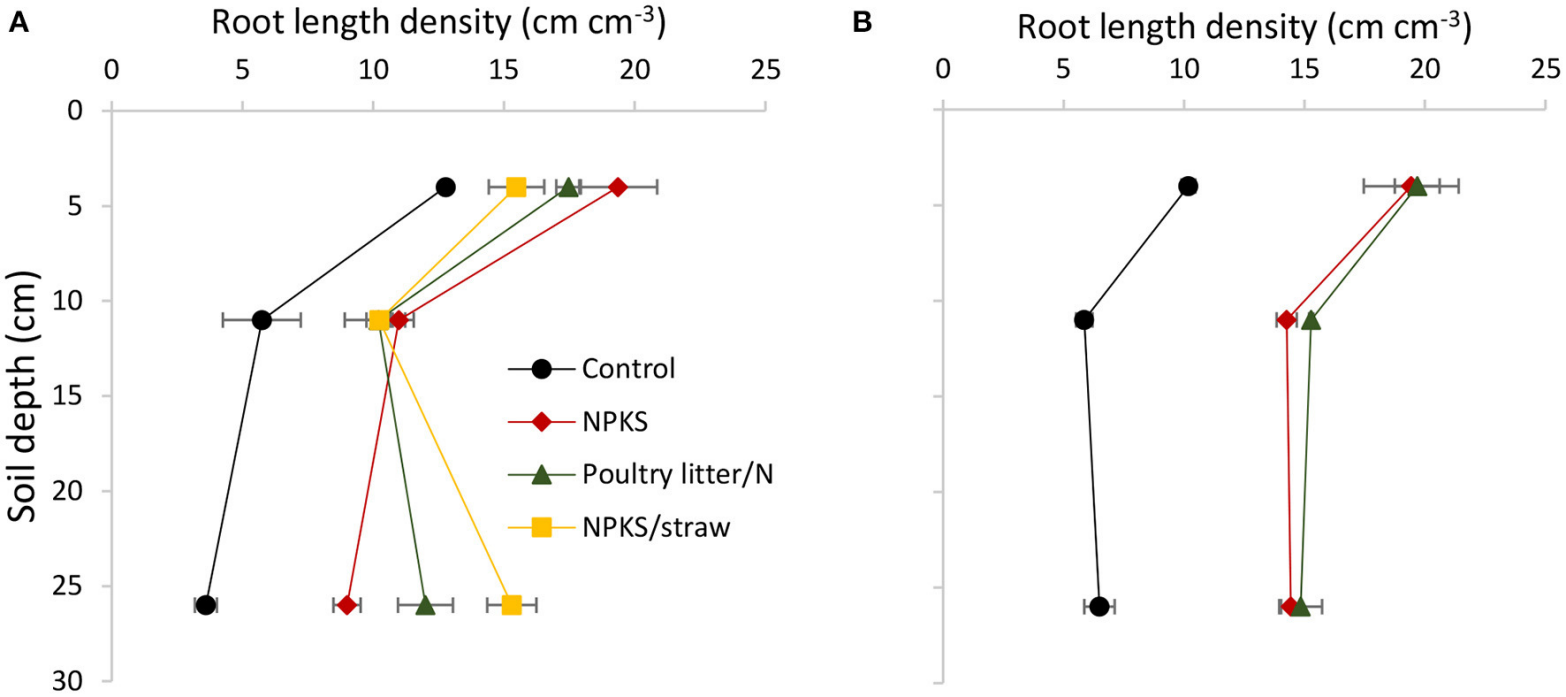
Effect of different amendments on the root length density of wheat plants in the topsoil (0–8 cm), the amendment (8–14 cm), and the subsoil (14–38 cm) layer in a Sodosol **(A)** and Vertosol **(B)**. Error bars represent means ± the SE of means of three replicates.

Subsoil amendments resulted in significantly (*p*< *0.0*5) higher root density in all layers, relative to the control in both Sodosol and Vertosol (Figures 1A,B). Root length density did not differ among different amendments in the amendment layer. However, the straw/NPKS and poultry litter/N produced 3.3-fold and 2.3-fold increase in root densities, respectively, relative to the control in the subsoil in Sodosol. When compared with NPKS, all organic amendments increased root length density in the subsoil in Sodosol, but not in Vertosol.

### Soil Moisture and Gas Exchange Measurements for the Youngest Wheat Leaves

**Figure 2:**
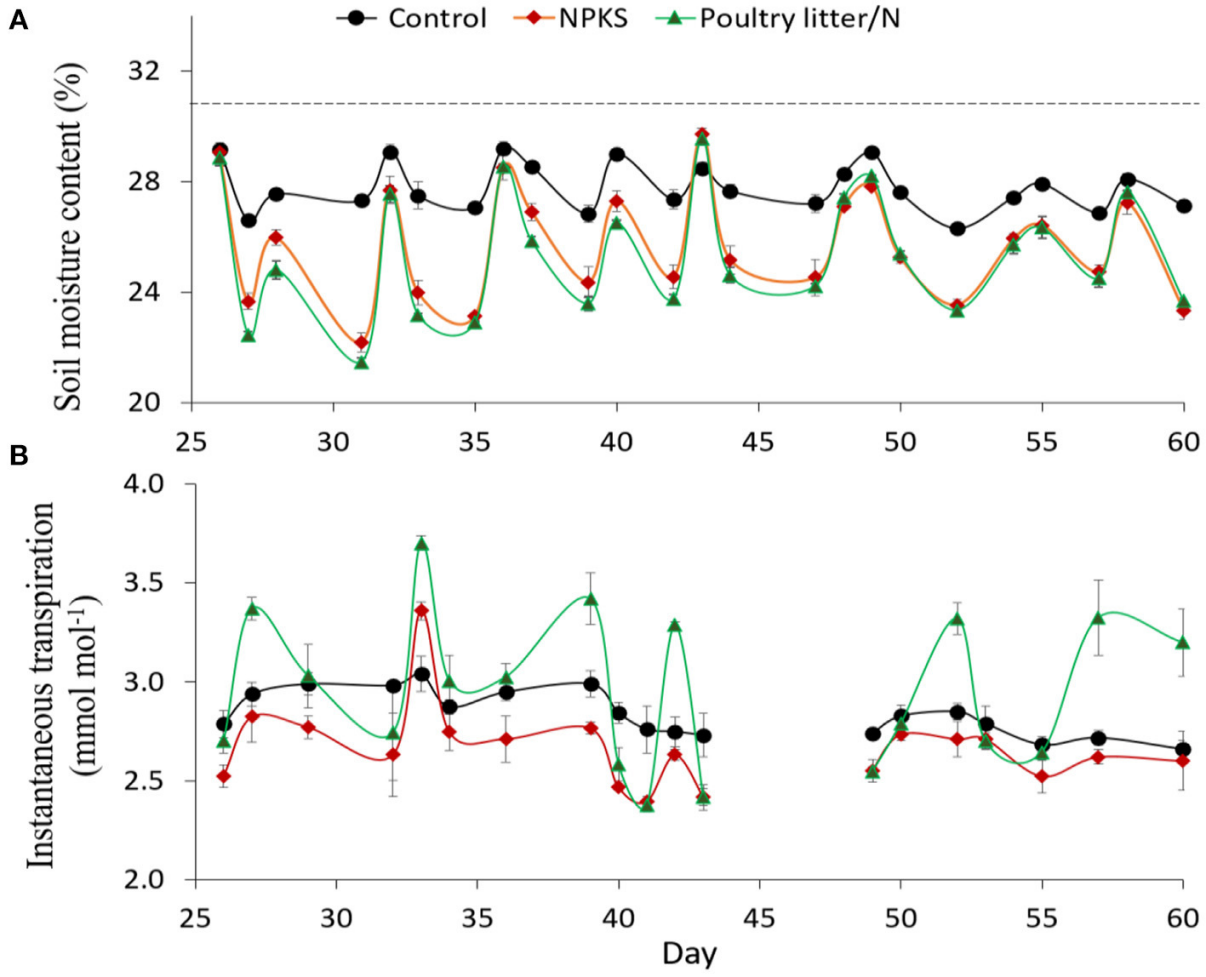
The soil moisture content of the Sodosol soil before each watering event **(A)**, and instantaneous leaf transpiration efficiency (TE) **(B)** 2 h after each watering event, for selected treatments in Experiment 2. Error bars represent means ± the SE of three replicates. The dotted line represents the targeted soil moisture content by each watering event. (Note: the soil columns were watered every 1 or 2 days).

Soil moisture content calculated before each watering event in the Sodosol in Experiment 2 fluctuated markedly between days 26 and 60 in line with the regular soil drying and re-watering cycles (Figure 2A). The largest fluctuations, indicating large volumes of soil water being extracted by the wheat plants, occurred with the PL/N and NPKS amendments while the control showed the smallest fluctuations. Noticeably, water extracted by wheat plants for the PL treatment matched that of the PL/N and NPKS up to day 30 but then decreased over time as plants became more N deficient.

The instantaneous leaf TE measured 2 h after re-watering the Sodosol soil columns, varied among the selected amendment treatments over time (Figure 2B). Noticeably, the spikes in instantaneous leaf TE between days 26 and 60 coincided with the times when the lowest soil moisture contents were recorded prior to re-watering (Figures 2A,B). Thus, increased leaf TE, relative to the control and NPKS, occurred with the PL/N amendments on days when the pre-watering moisture content dropped below 24%. For the NPKS amendment, the instantaneous leaf TE was less related to the pre-watering soil moisture content and was relatively lower or similar to that of the control (Figure 2B). In contrast, the instantaneous leaf TE did not differ between NPKS and PL/N in Vertosol (data not shown).

The effect of amendment treatments on gas exchange measurements and instantaneous TE also varied with the watering and drying cycles and was particularly affected by post-watering soil water status (Table 3). For example, on day 24, 12 h after watering, when wheat plants were not water-stressed, there were no differences in the stomatal conductance or in transpiration between the amendments. The PL, PL/N, and straw/NPKS amendments resulted in higher CO_2_ assimilation rates than the NPKS treatment, but there were no differences in leaf TE between these amendments. However, moderate water stress resulted in higher instantaneous leaf TE for the PL/N and straw/NPKS than for the NPKS treatment (Table 3). This moderate water stress, invoked by withholding the watering for 28 h on day 39, gave rise to the mild wilting of the lower leaves for treatments such as NPKS (Table 3) and resulted in larger reductions in the stomatal conductance and transpiration rates for the PL/N and straw/NPKS relative to other treatments. Leaf CO_2_ assimilation rates also decreased sharply, relative to control and well-water plants, but did not vary among all amendments.

**Table 3:** Leaf CO_2_ assimilation rate (μmol CO_2_ m^−2^ s^−1^).

**Amendment**	**Well-watered**				**Moderate water stress**				**Post-stress recovery**			
	**(12 h after watering)**				**(Without watering** **>28 h)**				**(2 h after watering)**			
	**(Early tillering)**				**(Stem elongation)**				**(Stem elongation)**			
	**A**	**g_s_**	**E**	**TE**	**A**	**g_s_**	**E**	**TE**	**A**	**g_s_**	**E**	**TE**
Control	15.9 a	0.59 a	5.68 a	2.74	16.2 b	0.46 c	5.21 c	3.11 a	15.9	0.49 ab	5.51 c	2.88 a
Poultry litter	17.9 b	0.86 b	6.21 b	2.88	12.4 a	0.16 b	2.91 b	4.25 bc	16.9	0.58 b	5.29 bc	3.21 b
Poultry litter/N	17.8 b	0.83 b	6.30 b	2.75	11.2 a	0.10 a	2.37 a	4.47 c	15.8	0.35 a	4.60 a	3.43 b
NPKS	16.2 a	0.78 b	6.18 b	2.62	12.3 a	0.14 ab	2.97 b	4.14 b	16.9	0.84 c	6.13 d	2.77 a
NPKS/straw	17.8 b	0.84 b	6.32 b	2.82	11.2 a	0.11 a	2.47 a	4.54 c	15.8	0.44 ab	4.97 ab	3.19 b

*(A), stomatal conductance (mol m^−2^s^−1^) (g_s_), transpiration rate (mmol H_2_O m^−2^s^−1^) (E), transpiration rate (mmol H_2_O m^−2^s^−1^) (E) and TE (mmol mol^−1^) of the youngest fully expanded leaf of wheat plants grown for 62 days in Sodosol under well-watered, moderate water stress and during the post-stress recovery period in Experiment 2. Different letters indicate significant differences (p < 0.05) between treatments, using Duncan's multiple range test*.

A similar pattern with the gas exchange measurements occurred during the post-stress recovery period (Table 3). Two hours after re-watering, the stomatal conductance for the NPKS amendment had recovered to that of well-watered plants, but the conductance remained lower in the PL/N, straw/NPKS, and PL leaves. Consequently, the instantaneous leaf TE was significantly higher with the PL, PL/N, and the straw/NPKS compared to that for the NPKS treatment during this post-stress recovery period.

### Bacterial Communities in Subsoils Below the Amendment Bands

**Figure 3:**
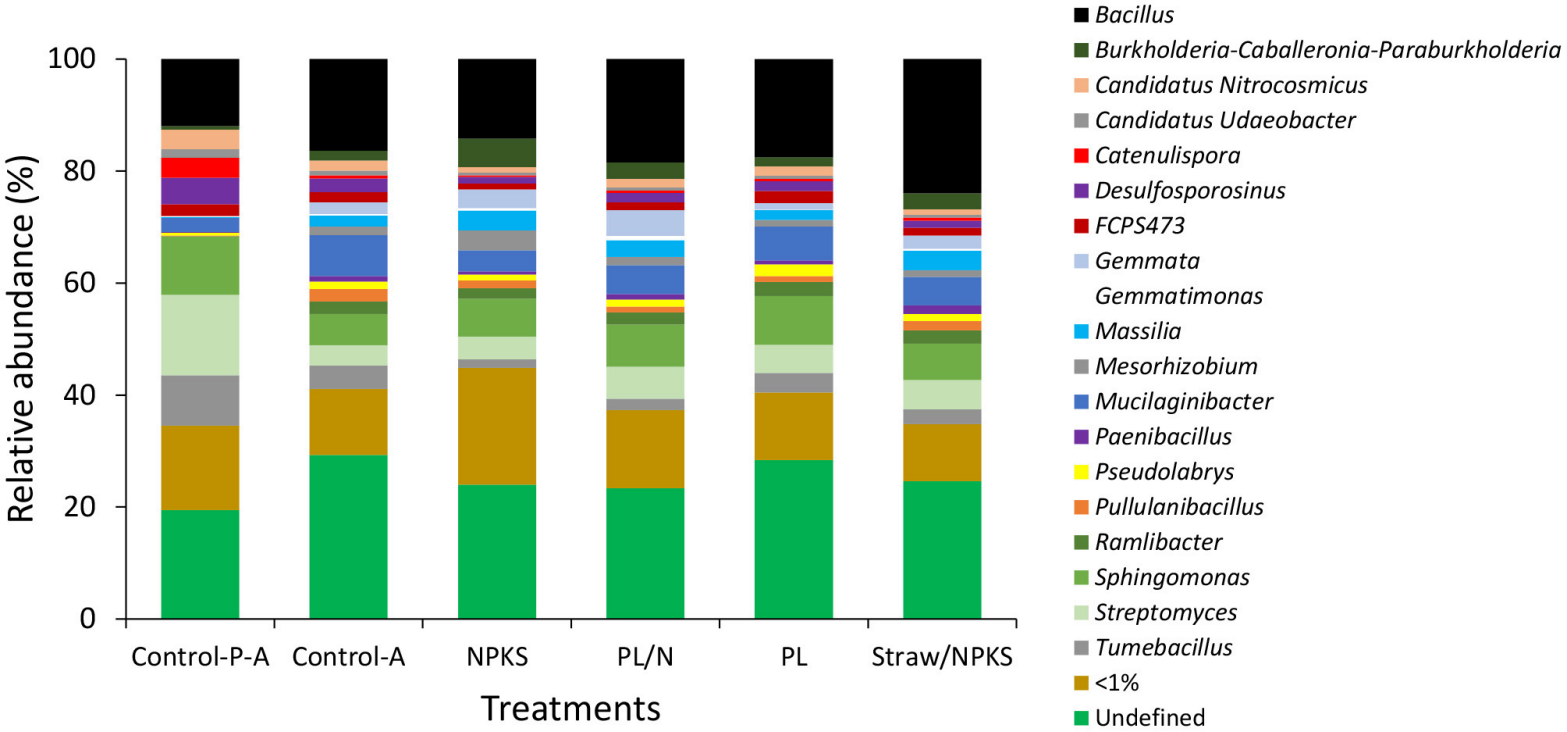
Relative abundance of bacterial genera in Sodosol subsoils collected below the amendment band in Experiment 2. The treatments include no amendments and no plants (Control-P-A), no amendments with plants (Control-A), and subsoils amended with chemical fertilizers (NPKS), NPKS plus wheat straw (NPKS/straw), poultry litter (PL), and poultry litter plus N (PL/N) in Experiment 2. (<1% means individual bacterial genera whose relative abundance is <1%).

The structure of the bacterial communities, at the genus level, was greatly affected by the presence of plant roots and amendments (Figure 3). While the *Streptomyces* genus was dominating in the unplanted soil, *Bacillus* was the dominant genus in the planted soil, reaching more than 15% of the total genera. When compared with the NPKS, the relative abundance of *Bacillus* increased by 69 and 30% with the straw/NPKS and PL/N, respectively.

The impact of deep-banded amendments on the bacterial community structure was further indicated by the differences in β-diversity in the NMMD plots (Supplementary Figure 1). There was a clear separation between the bacterial communities in the Sodosol subsoils without plant roots and amendments, amended with NPKS and the organic-based PL, PL/N, and straw/NPKS. In contrast, the diversity and structure of the bacterial communities in the Vertosol subsoils were not affected by the presence of plant roots or amendments such as PL/N and NPKS.

**Figure 4:**
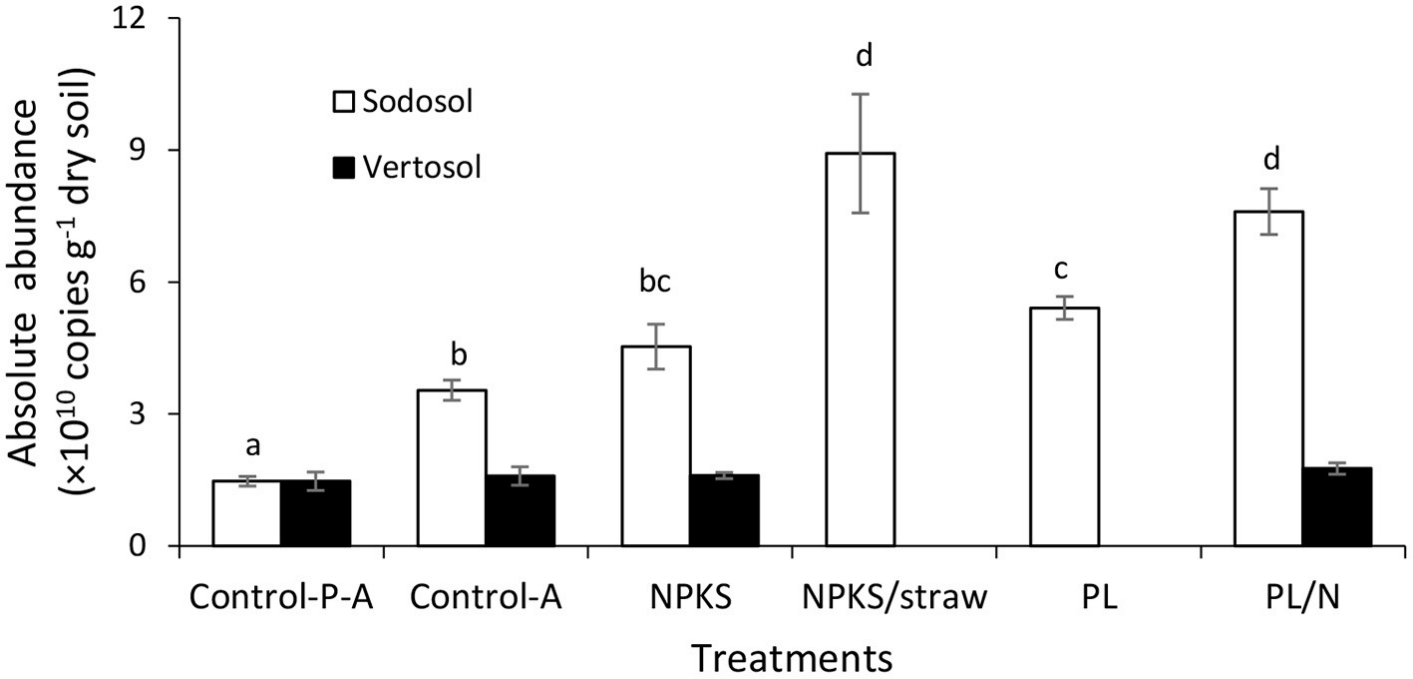
The absolute abundance of *Bacillus* genus in subsoil of Sodosol and Vertosol, collected below the amendment bands, following the growth of wheat plants for 62 days in Experiment 2. The treatments include no plants with no amendments (Control-P-A), plants with no amendments (Control-A), and subsoils amended with NPKS, NPKS/straw, PL, and PL/N in Experiment 2. Error bars represent means ± the standard error of three replicates. Different letters indicate significant treatment differences (*p* < 0.05) identified using Duncan's multiple range test in Sodosol. There is no significant difference among treatments in Vertosol.

There were also marked differences in the absolute *Bacillus* abundance between amendment treatments in the Sodosol subsoils (Figure 4). The highest abundance occurred with the fertilizer-enriched organic amendments straw/NPKS, and the lowest with the NPKS and control. The absolute abundance of *Bacillus* spp. in the straw/NPKS-amended soil was more than double that with the NPKS amendment. The *Bacillus* abundance also increased by nearly 100% in the presence, relative to in the absence of plant roots. In contrast, the relative abundance of *Bacillus* in Vertosol was <2.5% and there were no differences in the relative and absolute *Bacillus* abundances in the subsoils between the control, the PL/N, and the NPKS treatments (Figure 4).

**Figure 5:**
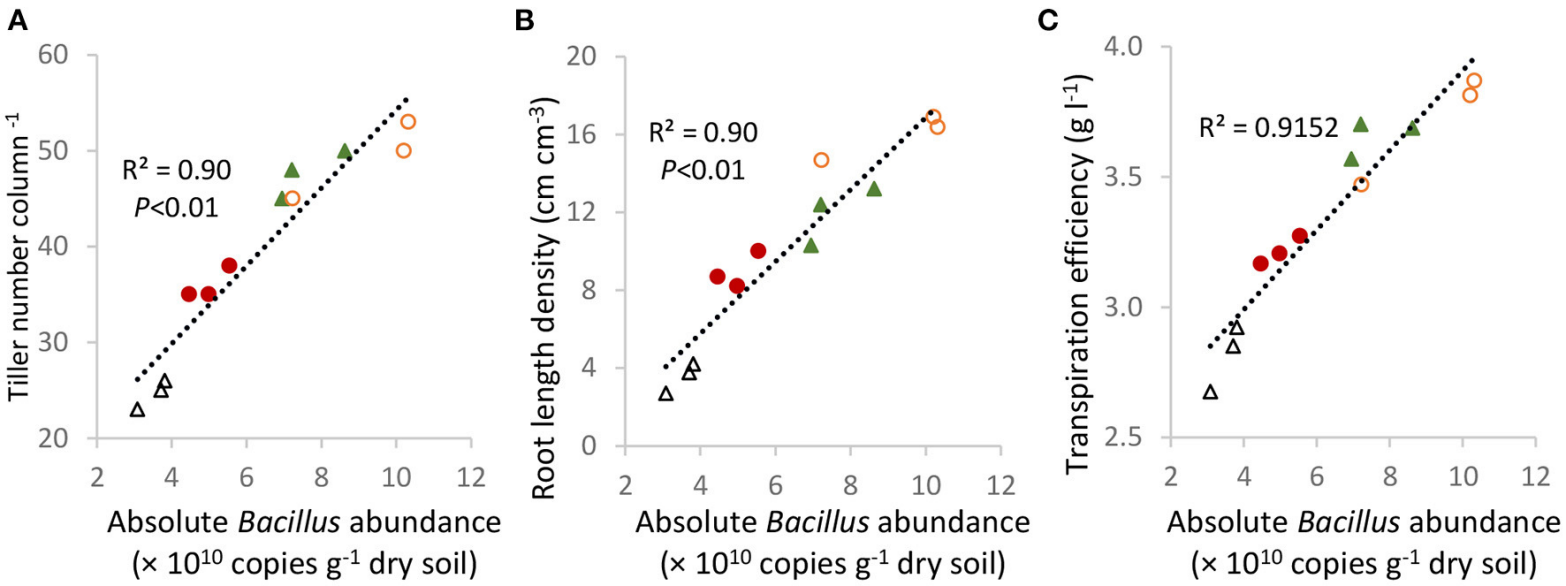
Significant linear relationships between tiller numbers at day 35 **(A)**, root length density **(B)**, and canopy TE values **(C)** were measured over 62 days, with the absolute *Bacillus* abundance in the Sodosol subsoils in Experiment 2. Symbols identify the treatments control (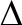), NPKS amendment (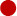), NPKS/straw (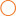), and PL/N (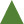).

**Figure 6:**
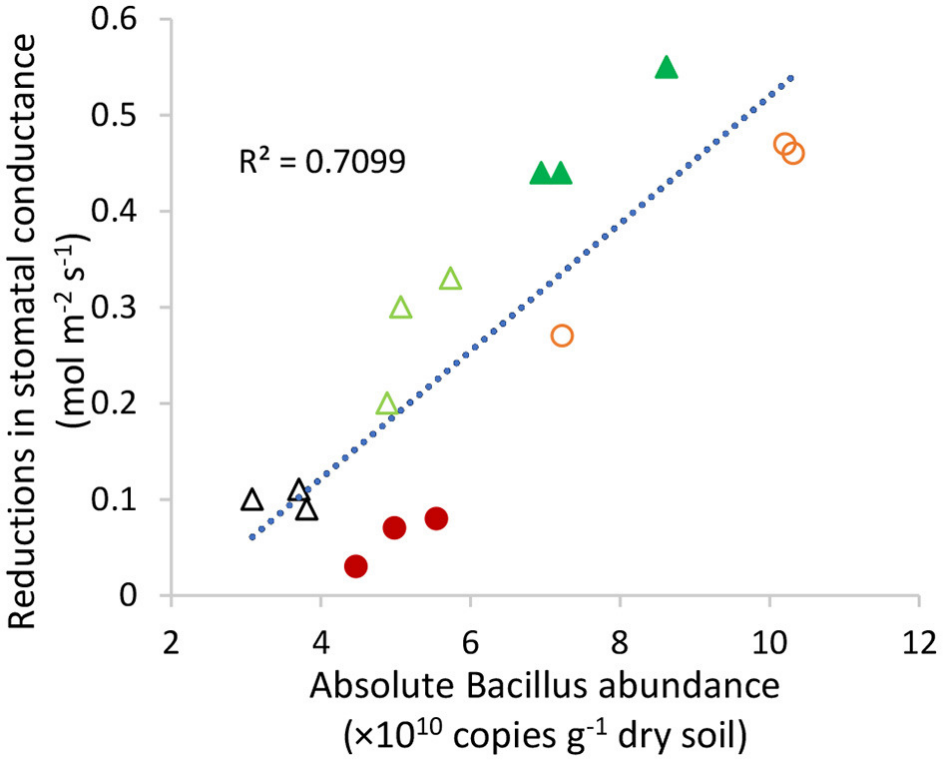
Correlation between reductions in stomatal conductance at the post-stress recovery period (day 39) and the absolute abundance of *Bacillus* in the Sodosol subsoils in Experiment 2. Symbols identify the treatments control (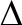), NPKS amendment (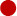), NPKS/straw (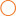), PL/N (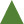), and PL (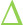).

Highly significant linear relationships were detected between tiller numbers at day 35 (Figure 5A) (*R*^2^ = 0.90, *p* < 0.01), root length density (Figure 5B) at day 62 (*R*^2^ = 0.90, *p* < 0.01), and the canopy TE measured over the 62 days (Figure 5C) (*R*^2^ = 0.92, *p* < 0.01) with the absolute abundance of *Bacillus* in the Sodosol subsoil. Moreover, the absolute abundance of *Bacillus* sp. showed significant positive correlations with reductions in stomatal conductance at the post-stress recovery stage (Figure 6).

## Discussion

### Effect of Organic Amendments on TE

The two experiments confirmed that the canopy TE, a previously considered constant property for a given crop in a given environment, can be increased by the deep banded nutrient-rich organic amendments to a Sodosol. The response was quite substantial with shoot-TE values for the nutrient-enriched organic amendments exceeding the control and inorganic NPKS by an average of 44 and 23%, respectively, at stem elongation in Experiment 1, and by 30 and 15% at the booting stage in Experiment 2. These increases are comparable to the shoot-TE increases of 33 and 45% for pasture grasses (Espinosa et al., 2011) and winter wheat (Wang et al., 2017), respectively, following the addition of manure in other studies. In this study, there was some process associated with the use of organic amendments that consistently invoked vigorous plant growth, e.g., higher tiller number and greater biomass accumulation, but not excessive transpiration and water loss from crop canopy. In alignment with an increase in canopy TE, instantaneous leaf TE also increased with organic amendments under moderate water stress or during the post water-stress recovery. The nature of the underlying mechanisms responsible for these beneficial outcomes needs to be understood.

### The Physiological Basis for the TE Response

Increases in instantaneous TE by the addition of nutrient-enriched organic amendments were attributed to greater reductions in stomatal conductance and transpiration rates under moderate water stress or during the post-water recovery period. When moderate water stress was invoked by withholding water for more than 28 h at the late growth stage, there were decreases in stomatal conductance and increases in TE for all treatments including the control. This aligns with a well-known finding that plant roots are capable of sensing dry soil and respond by synthesizing hormones to induce stomatal closure (Saradadevi et al., 2017; Brilli et al., 2019). However, plants grown in the PL/N and straw/NPKS-amended Sodosol showed greater reductions in stomatal conductance and transpiration rate than in NPKS-amended soil regardless of being subjected to similar water stress. Even after re-watering, leaf TE was still higher in the presence of PL/N and straw/NPKS, relative to NPKS, due to a delayed recovery in the stomatal conductance. Importantly, the mechanism that invoked the TE increases with the organic amendments by reducing stomatal conductance during the post-water recovery period did not impair the ability of the plants to assimilate CO_2_. There is evidence that CO_2_ diffusion into a stomatal cavity was less inhibited by stomatal closure than water loss by transpiration from the cavity (Sikuku et al., 2010; Jones, 2014). Possibly, other signaling mechanisms, in addition to water stress between the roots and shoot stomata, have been operating in affecting TE in Sodosol amended with PL/N and straw/NPKS.

The nutrient-enriched organic amendments also resulted in higher photosynthetic rates at early growth stages when an adequate plant water status was maintained. This points to a growth-promoting effect of the organic amendments relative to standard inorganic fertilizer. Higher photosynthetic rates are consistent with the higher early tillering, shoot biomass, and pronounced root proliferation in the organic-amended sodic subsoil compared to the NPKS treatments. The positive effect of the organic amendments on plant photosynthetic rates had also been reported for pears (Xu et al., 2001), rice (Halim et al., 2018), and wheat (Wang et al., 2017), and was likely attributed to their fertilizer, particularly, their N effect (Celestina et al., 2019). However, in this study, soil N availability was in no way responsible for the higher canopy TE that occurred with the PL, PL/N, and straw/NPKS relative to NPKS treatment. Indeed, wheat plants grown in PL-amended soil showed severe N-deficiency symptoms by the stem elongation growth stage, but still recorded a higher canopy TE than that of NPKS. Straw/NPKS also conferred a higher TE, relative to NPKS, yet the shoot N concentration was 29% lower than the NPKS treatment due presumably to a straw-induced microbial N immobilization and low soil N availability. Therefore, it is the organic nature of the amendment themselves, not the nutritional effect, that is responsible for their growth-promoting effect.

### The Role of *Bacillus* sp. rhizobacteria

The addition of nutrient-enriched organic amendments resulted in a significant increase in the abundance of *Bacillus*, a genus containing many species of plant beneficial rhizobacteria. In contrast, the inorganic NPKS amendment showed an apparent suppressive effect on the relative abundance of *Bacillus* sp., compared to the control. This is aligned with previous findings that organic fertilization could promote specific groups of plant beneficial microbial taxa, relative to the chemical fertilizers (Francioli et al., 2016; Tan et al., 2019). Moreover, the presence of wheat roots significantly increased the abundance of *Bacillus* sp. compared to the unplanted control, indicating that the plant rhizosphere and associated root exudates might provide a favorable environment for these bacteria (Olanrewaju et al., 2019). Thus, the highest root length densities detected for the straw/NPKS and PL/N treatments resulted in the highest absolute abundance of *Bacillus* sp. in the subsoil. It is likely that these nutrient-enriched organic amendments stimulated the localized root proliferation in and around the amendment band, and consequently, led to the high levels of rhizosphere colonization by the *Bacillus* sp. in Sodosol.

The greater response of *Bacillus* to straw/NPKS and PL/N than NPKS might account for the increases in the canopy and leaf TE in the Sodosol. This is supported by two findings from this study. First, there was a strong positive correlation (*r*^2^ = 0.91; *p* < 0.01) between the canopy TE and the absolute abundance of *Bacillus* in the Sodosol subsoil. Second, the reductions in stomatal conductance, which provides the physiological basis for decreases in transpiration rate and increases in leaf TE under water-stressed conditions, were also closely correlated with the abundance of *Bacillus*. Other studies also found that inoculation of plant roots with *Bacillus* spp. could mitigate water stress in plant species such as common bean, maize, and pepper *via* a decrease in stomatal conductance (Li et al., 2016; Samaniego-Gámez et al., 2016; de Lima et al., 2019). The effect of *Bacillus* on reducing stomatal conductance and transpiration during or after the moderate water stress, therefore, possibly contributed to more efficient water use or a “water-saving” outcome by the organic amendments in this study.

The enhanced plant biomass with the organic-based amendments, relative to inorganic fertilizer, might have also been attributed to increases in *Bacillus* abundance. Direct evidence comes from the significant positive correlation (*r*^2^ = 0.90, *p* < 0.01) between tiller numbers and *Bacillus* abundance in the subsoil. Also, the nutrient-rich organic amendments which led to high *Bacillus* abundance in the subsoil also resulted in higher CO_2_ assimilation rates under well-watered conditions. There is a substantial body of evidence showing how plant beneficial bacteria such as *Bacillus* spp. were able to trigger the synthesis of plant growth-promoting substances and increase leaf photosynthetic rates (Xie et al., 2014; Gagné-Bourque et al., 2016; Radhakrishnan and Lee, 2016; Barnawal et al., 2017). On the other hand, wheat plants grown in the Sodosol, amended with organic amendments, appeared to be more tolerant to water stress as wilting of the lower leaves was delayed or never observed relative to NPKS treatment. The maintenance of the leaf water content should alleviate the adverse effects of water stress on leaf photosynthesis and biomass production. This is also in line with previous findings that plants inoculated with *Bacillus subtilis* could maintain a higher leaf water status and photosynthetic activity (Ahmad et al., 2013; Barnawal et al., 2017; de Lima et al., 2019).

Thus, the basis for increased canopy TE with the organic-based amendments becomes apparent. It resulted from the enhanced photosynthetic rates under well-watered conditions together with the water-saving effects *via* reductions in stomatal conductance during and following periods of moderate water stress. Both of these effects could be linked to increases in the colonization of the wheat roots by *Bacillus*. Particularly, the *Bacillus*-regulation of stomatal conductance would lead to better plant growth and resilience when water stress has occurred. The apparent effect of *Bacillus* on stomatal conductance had been attributed to the production and release of abscisic acid (ABA), a key chemical signaling agent involved in stomatal regulation (Kumar et al., 2012; Samaniego-Gámez et al., 2016). Further studies are required to understand the complex link between organic amendments, the soil microbiome, leaf hormones, plant growth responses and canopy TE.

### The Role of Improved Subsoil Structure

The direct and indirect effects of the organic amendments on the physical environment in the Sodosol subsoil might have partly contributed to the increases in canopy TE. Poor aeration and high soil strength associated with Sodosol subsoils often restrict plant root growth and limit access of the plants to subsoil nutrients and water (MacEwan et al., 2010). The presence of pore space within the organic amendment bands in the subsoil would facilitate the initial root access to the nutrients in the amendments, which would explain a better shoot growth and biomass production at the early growth stage, relative to NPKS treatment. Moreover, our previous study found that the deep placement of nutrient-rich organic amendments, such as straw/NPKS and PL/mac, could enhance deep root proliferation and root-associated bacterial and fungal abundance, resulting in large increases in the percentage of large (>2,000 μm) water-stable macroaggregates in the subsoil (Wang et al., 2020). It follows that wheat plants grown in organic-amended soils would proliferate more easily and/or take up water and nutrients more readily due to improved physical structure. This meant that less carbohydrates would be consumed by roots in penetrating the dense, poorly aerated clay subsoil.

Further insights into soil physical conditions that invoked the increase in canopy TE can be gained by comparing the amendment effect in the Vertosol and Sodosol. The Vertosol is characterized by a well-structured subsoil with an air-filled porosity of 26% at field capacity, which was more than twice that in the Sodosol subsoil (12%) (Wang et al., 2019). Earlier studies provided evidence that soil structure, such as the proportion of macroaggregates or aeration, is a key driver in shaping the microbial community structure in subsoils (Turner et al., 2017; Celestina et al., 2019). Thus, the improved aeration in the Sodosol following the addition of organic amendments was likely to have facilitated the initial colonization of the wheat roots by *Bacillus*. In contrast, there were little changes in the abundance of *Bacillus* and bacterial diversity in response to the presence of plant roots or organic amendments in the Vertosol, which might be related to its favorable physical condition within the well-structured subsoil. Similarly, the higher shoot biomass and canopy TE of wheat plants in the Vertosol, relative to the Sodosol, might also be associated with the favorable physical structure in Vertosol subsoil and consequently, showed no response to PL/N relative to NPKS amendments.

## Conclusion

The deep application of nutrient-enriched organic amendments consistently increased the canopy TE of wheat plants grown in Sodosol compared with inorganic fertilizer additions. The increased TE was largely a function of a similar or reduced water use and generally improved shoot biomass with these organic amendments. The positive effect of these organic amendments on canopy TE did not result from any increase in N supply given that leaf N showed no correlation with canopy TE. A marked increase in the absolute abundance of *Bacillus* species in the subsoil, close to the amendment band, occurred in the presence of deep-banded fertilizer-enriched organic amendments. This increase in the abundance of *Bacillus* spp. provides a possible explanation for the increases in wheat biomass, and for greater reductions in stomatal conductance and increases in leaf TE under moderate water stress occurred during the watering cycles. The effect of organic amendment on the physical structure of Sodosol subsoil might have also contributed to the increases in canopy TE. It is difficult to separate these two factors which might be equally important or interactively affect TE in the Sodosol. Further investigations are required to determine whether these benefits of nutrient-rich organic amendments on canopy TE in Sodosol soils occur under field conditions and with different crop species.

## Data Availability Statement

The original contributions presented in the study are publicly available. This data can be found here: National Center for Biotechnology Information (NCBI) BioProject database under accession number PRJNA736532.

## Author Contributions

XW, PS, and CT planned the experiment. XW set up the experiment and monitored the canopy gas exchange system. JJ and CK performed the soil microbial data analysis. XW and PS drafted the manuscript. JJ, CK, AF, RA, and CT revised the manuscript. All the authors read and approved the final manuscript.

## Funding

This research was supported under Grains Research and Development Corporation Projects funding scheme (project DAV00149).

## Conflict of Interest

The authors declare that the research was conducted in the absence of any commercial or financial relationships that could be construed as a potential conflict of interest.

## Publisher's Note

All claims expressed in this article are solely those of the authors and do not necessarily represent those of their affiliated organizations, or those of the publisher, the editors and the reviewers. Any product that may be evaluated in this article, or claim that may be made by its manufacturer, is not guaranteed or endorsed by the publisher.

## References

[B1] Ahmad M. Zahir Z. A. Khalid M. Nazli F. Arshad M. (2013). Efficacy of *Rhizobium* and *Pseudomonas* strains to improve physiology, ionic balance and quality of mung bean under salt-affected conditions on farmer's fields. Plant Physiol. Biochem. 63, 170–176. 10.1016/j.plaphy.2012.11.024 23262185

[B2] Akhtar S. S. Amby D. B. Hegelund J. N. Fimognari L. Großkinsky D. K. Westergaard J. C.. (2020). *Bacillus licheniformis* FMCH001 increases water use efficiency *via* growth stimulation in both normal and drought conditions. Front. Plant Sci. 11:297. 10.3389/fpls.2020.00297 32318078 PMC7155768

[B3] Barnawal D. Bharti N. Pandey S. S. Pandey A. Chanotiya C. S. Kalra A. (2017). Plant growth-promoting rhizobacteria enhance wheat salt and drought stress tolerance by altering endogenous phytohormone levels and TaCTR1/TaDREB2 expression. Physiol. Plant. 161, 502–514. 10.1111/ppl.12614 28786221

[B4] Brilli F. Pollastri S. Raio A. Baraldi R. Neri L. Bartolini P.. (2019). Root colonization by *Pseudomonas chlororaphis* primes tomato (*Lycopersicum esculentum*) plants for enhanced tolerance to water stress. J. Plant Physiol. 232, 82–93. 10.1016/j.jplph.2018.10.029 30537616

[B5] Cabrera-Bosquet L. Molero G. Bort J. Nogués S. Araus J. L. (2007). The combined effect of constant water deficit and nitrogen supply on WUE, NUE and Δ^13^C in durum wheat potted plants. Ann. Appl. Biol. 15, 277–289. 10.1111/j.1744-7348.2007.00195.x

[B6] Callahan B. J. McMrdie P. J. Rosen M. J. Han A. W. Johnson A. J. A. Holmes S. P. (2016). DADA2: High-resolution sample inference from Illumina amplicon data. Nat. Methods 13, 581–583. 10.1038/nmeth.3869 27214047 PMC4927377

[B7] Caporaso J. G. Lauber C. L. Walters W. A. Berg-Lyons D. Huntley J. Fierer N.. (2012). Ultra-high-throughput microbial community analysis on the Illumina HiSeq and MiSeq platforms. ISME J. 6, 1621–1624. 10.1038/ismej.2012.8 22402401 PMC3400413

[B8] Celestina C. Wood J. L. Manson J. B. Wang X. Sale P. W. Tang C.. (2019). Microbial communities in top-and subsoil of repacked soil columns respond differently to amendments but their diversity is negatively correlated with plant productivity. Sci. Rep. 9, 1–12. 10.1038/s41598-019-45368-9 31222122 PMC6586782

[B9] Condon A. G. Richards R. Rebetzke G. Farquhar G. (2002). Improving intrinsic water-use efficiency and crop yield. Crop Sci. 42, 122–131. 10.2135/cropsci2002.1220 11756262

[B10] de Lima B. C. Moro A. L. Santos A. C. P. Bonifacio A. Araujo A. S. F. de Araujo F. F. (2019). *Bacillus subtilis* ameliorates water stress tolerance in maize and common bean. J. Plant Interact. 14, 432–439. 10.1080/17429145.2019.1645896

[B11] de Wit C. (1958). Transpiration and crop yields. Institute of biological and chemical research on field crops and herbage. The Netherlands: Wageningen, 1–88.

[B12] Denman S. E. McSweeney C. S. (2006). Development of a real-time PCR assay for monitoring anaerobic fungal and cellulolytic bacterial populations within the rumen. FEMS Microbiol. Ecol. 58, 572–582. 10.1111/j.1574-6941.2006.00190.x 17117998

[B13] Ehlers W. Goss M. (2016). Water dynamics in plant production. Wallingford: CABI Publishing.

[B14] Espinosa D. Sale P. W. Tang C. (2011). Changes in pasture root growth and transpiration efficiency following the incorporation of organic manures into a clay subsoil. Plant Soil 348, 329–343. 10.1007/s11104-011-0951-3

[B15] Faralli M. Matthews J. Lawson T. (2019). Exploiting natural variation and genetic manipulation of stomatal conductance for crop improvement. Curr. Opin. Plant Bio. 49, 1–7. 10.1016/j.pbi.2019.01.003 30851622 PMC6692497

[B16] Francioli D. Schulz E. Lentendu G. Wubet T. Buscot F. Reitz T. (2016). Mineral vs. organic amendments: microbial community structure, activity and abundance of agriculturally relevant microbes are driven by long-term fertilization strategies. Front. Microbiol. 7:1446. 10.3389/fmicb.2016.01446 27683576 PMC5022044

[B17] Gagné-Bourque F. Bertrand A. Claessens A. Aliferis K. A. Jabaji S. (2016). Alleviation of drought stress and metabolic changes in timothy (*Phleum pratense* L.) colonized with *Bacillus subtilis* B26. Front. Plant Sci. 7:584. 10.3389/fpls.2016.00584 27200057 PMC4854170

[B18] Halim A. Sa'adah N. Abdullah R. Karsani S. A. Osman N. Panhwar Q. A.. (2018). Influence of soil amendments on the growth and yield of rice in acidic soil. Agron 8:165. 10.3390/agronomy8090165

[B19] Isbell R. (2016). The Australian soil classification. Wallingford: CSIRO publishing.

[B20] Jones H. G. (2014). Plants and microclimate: a quantitative approach to environmental plant physiology. Cambridge: Cambridge University Press.

[B21] Kondo M. Ideta O. Barlaan E. Imbe T. Itoh S. Pablico P.. (2004). Genotypic variations of δ ^13^C in rice (*Oryza sativa* L. and *Oryza glaberrima* Steud.) in relation to transpiration efficiency and biomass production as affected by soil water conditions and N. Proc. 4th Inter. Crop Sci. Cong., Brisbane, Australia.

[B22] Kumar A. S. Lakshmanan V. Caplan J. L. Powell D. Czymmek K. J. Levia D. F.. (2012). Rhizobacteria *Bacillus* subtilis restricts foliar pathogen entry through stomata. Plant J. 72, 694–706. 10.1111/j.1365-313X.2012.05116.x 22862801

[B23] Li Y. Xu S. Gao J. Pan S. Wang G. (2016). Bacillus subtilis-regulation of stomatal movement and instantaneous water use efficiency in Vicia faba. Plant Grow. Regul. 78, 43–55. 10.1007/s10725-015-0073-7

[B24] Lou J. Yang L. Wang H. Wu L. Xu J. (2018). Assessing soil bacterial community and dynamics by integrated high-throughput absolute abundance quantification. PeerJ. 6:e4514. 10.7717/peerj.4514 29576979 PMC5857175

[B25] MacEwan R. Crawford D. Newton P. Clune T. (2010). High clay contents, dense soils, and spatial variability are the principal subsoil constraints to cropping the higher rainfall land in south-eastern Australia. Soil Res. 48, 150–166. 10.1071/SR09076

[B26] Olanrewaju O. S. Ayangbenro A. S. Glick B. R. Babalola O. O. (2019). Plant health: feedback effect of root exudates-rhizobiome interactions. Appl. Microbiol. Biotechnol. 103, 1155–1166. 10.1007/s00253-018-9556-6 30570692 PMC6394481

[B27] Passioura J. (1996). Drought and drought tolerance. Plant Growth Regul. 20, 79–83. 10.1007/BF00024003

[B28] Passioura J. Angus J. (2010). Improving productivity of crops in water-limited environments. Adv Agron. 106, 37–75. 10.1016/S0065-2113(10)06002-5

[B29] Radhakrishnan R. Lee I. J. (2016). Gibberellins producing *Bacillus methylotrophicus* KE2 supports plant growth and enhances nutritional metabolites and food values of lettuce. Plant Physiol. Biochem. 109, 181–189. 10.1016/j.plaphy.2016.09.018 27721133

[B30] Rebetzke G. Condon A. G. Richards R. Farquhar G. (2002). Selection for reduced carbon isotope discrimination increases aerial biomass and grain yield of rainfed bread wheat. Crop Sci. 42, 739–745. 10.2135/cropsci2002.0739

[B31] Samaniego-Gámez B. Y. Garruña R. Tun-Suárez J. M. Kantun-Can J. Reyes-Ramírez A. Cervantes-Díaz L. (2016). *Bacillus* spp. inoculation improves photosystem II efficiency and enhances photosynthesis in pepper plants. Chil. J. Agric. Res. 76, 409–416. 10.4067/S0718-58392016000400003

[B32] Saradadevi R. Palta J. A. Siddique K. H. (2017). ABA-mediated stomatal response in regulating water use during the development of terminal drought in wheat. Front. Plant Sci. 8:1251. 10.3389/fpls.2017.01251 28769957 PMC5513975

[B33] Sikuku P. Netondo G. Onyango J. Musyimi D. (2010). Chlorophyll fluorescence, protein and chlorophyll content of three nerica rainfed rice varieties under varying irrigation regimes. ARPN J Agric Biol Sci. 5, 19–25. 10.3389/fpls.2015.00712 26442026 PMC4563168

[B34] Sinclair T. R. (2012). Is transpiration efficiency a viable plant trait in breeding for crop improvement? Funct. Plant Biol. 39, 359–365. 10.1071/FP11198 32480788

[B35] Stewart B. Lal R. (2018). Increasing world average yields of cereal crops: It's all about water. Adv. Agron. 151, 1–44. 10.1016/bs.agron.2018.05.001

[B36] Tan L. Gu S. Li S. Ren Z. Deng Y. Liu Z.. (2019). Responses of microbial communities and interaction networks to different management practices in tea plantation soils. Sustain 11:4428. 10.3390/su11164428

[B37] Turner S. Mikutta R. Meyer-Stüve S. Guggenberger G. Schaarschmidt F. Lazar C. S.. (2017). Microbial community dynamics in soil depth profiles over 120,000 years of ecosystem development. Front. Microbiol. 8:874. 10.3389/fmicb.2017.00874 28579976 PMC5437693

[B38] von Caemmerer S. Baker N. (2007). The biology of transpiration. from guard cells to globe. Plant Physiol. 143:3. 10.1104/pp.104.900213

[B39] Wang L. Wang S. Chen W. Li H. Deng X. (2017). Physiological mechanisms contributing to increased water-use efficiency in winter wheat under organic fertilization. PLoS ONE. 12:e0180205. 10.1371/journal.pone.0180205 28662113 PMC5491151

[B40] Wang X. J. Sale P. Hayden H. Tang C. Clark G. Armstrong R. (2020). Plant roots and deep-banded nutrient-rich amendments influence aggregation and dispersion in a dispersive clay subsoil. Soil Biol. Biochem. 141:107664. 10.1016/j.soilbio.2019.107664

[B41] Wang X. J. Sale P. Liu Z. Armstrong R. Rochfort S. Tang C. (2019). Allelopathic effects account for the inhibitory effect of field-pea (*Pisum sativum* L.) shoots on wheat growth in dense clay subsoils. Biol. Fertil. Soils. 55, 649–659. 10.1007/s00374-019-01384-5

[B42] WRB and IWG (2015). World reference base for soil resources 2014, update 2015: International soil classification system for naming soils and creating legends for soil maps: FAO:Rome, 192.

[B43] Xie S. S. Wu H. J. Zang H. Y. Wu L. M. Zhu Q. Q.. (2014). Plant growth promotion by spermidine-producing *Bacillus subtilis* OKB105. Mol. Plant Microbe Interact. 27, 655–663. 10.1094/MPMI-01-14-0010-R 24678831

[B44] Xu H. L. Wang X. Fujita M. (2001). Effects of organic farming practices on photosynthesis, transpiration and water relations, and their contributions to fruit yield and the incidence of leaf-scorch in pear trees. J. Crop. Prod. 3, 127–138. 10.1300/J144v03n01_12

[B45] Zhang Q. Ficklin D. L. Manzoni S. Wang L. Way D. Phillips R. P.. (2019). Response of ecosystem intrinsic water use efficiency and gross primary productivity to rising vapor pressure deficit. Enviro Res Lett. 14:074023. 10.1088/1748-9326/ab2603

