# Supplementary material for: An Insight Into the Effect of Organic Amendments on the Transpiration Efficiency of Wheat Plant in a Sodic Duplex Soil
Source: Front Plant Sci. 2021 Oct 20;12:722000. doi: 10.3389/fpls.2021.722000 (PMC8563830; doi:10.3389/fpls.2021.722000)
Supplement: Supplementary file 1 [file Data_Sheet_1.docx]

**Table S1.** Selected basic properties of the topsoil and subsoil for Sodosol and Vertosol soils used in the study.

| Soil Layer pH EC Organic C NO_3_^-^ +NH_4_^+^ Olsen P Clay Exchangeable Na  (0.01 M CaCl_2_) (dS m^-1^) (mg g^-1^) (µg N g^-1^) (µg g^-1^) (%) (% of cations) |
| --- |
| Sodosol Top 5.3 0.10 44.1 20.2 29.9 21.0 9.2  Sub 5.8 0.31 8.0 7.3 2.8 53.7 21.0  Vertosol Top 7.4 0.17 9.2 8.8 26.9 43.7 0.9  Sub 7.4 0.17 8.9 6.6 19.4 48.1 1.1 |


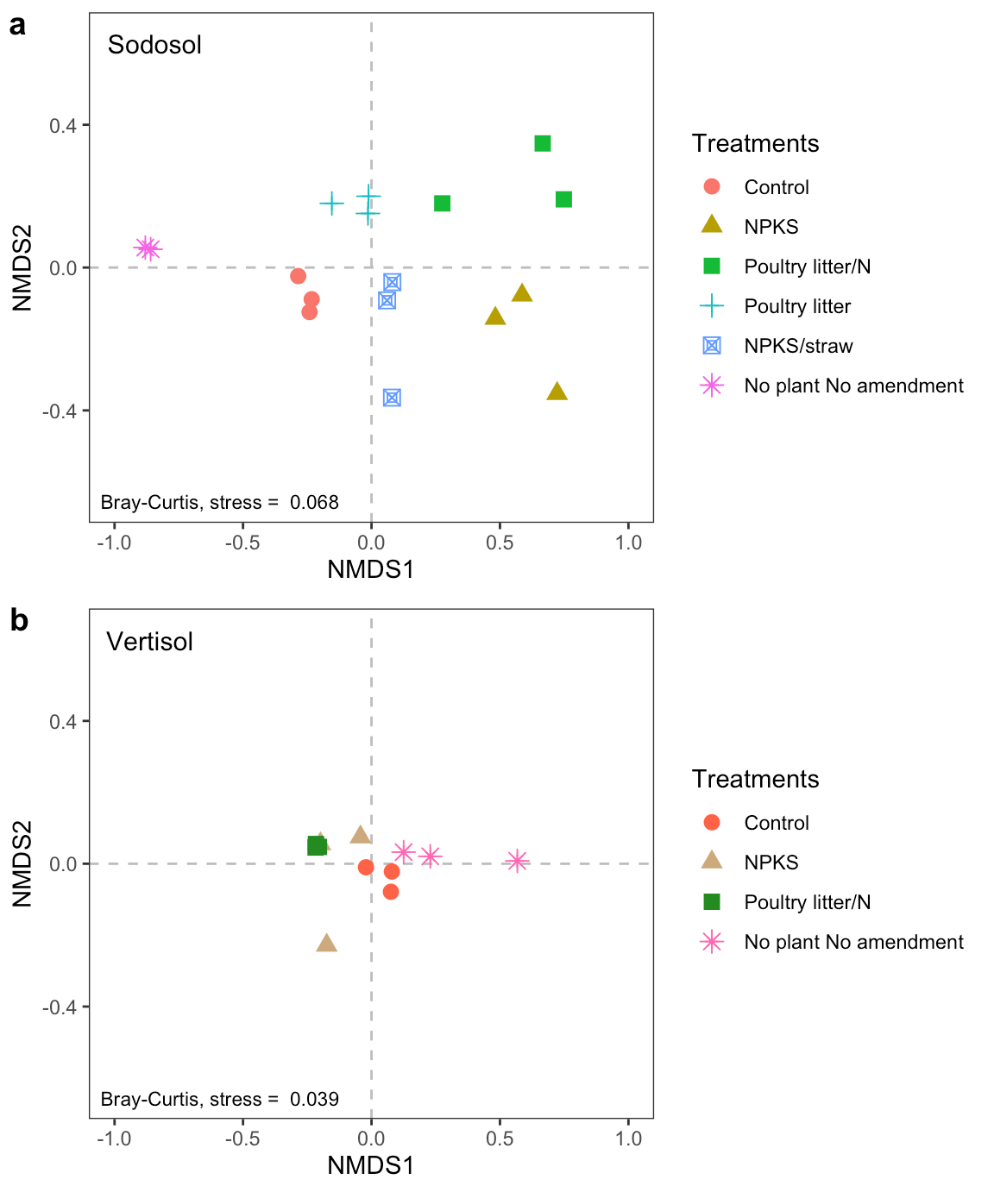

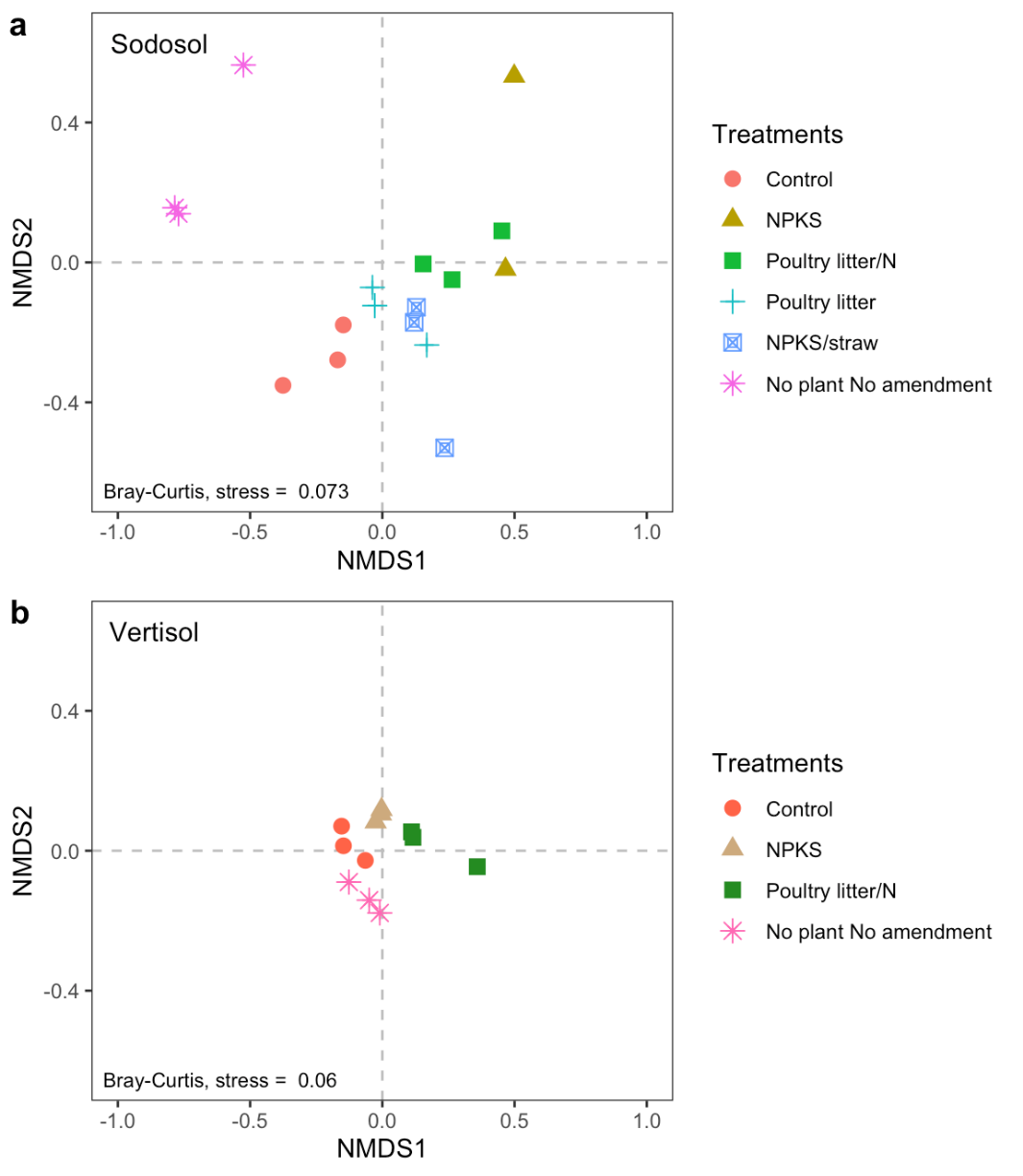


Vertosol

Sodosol

**Figure S1.** Comparison of β diversity by non-metric multidimensional scaling (NMDS) among different amendment treatments in the Sodosol (a) and Vertosol (b) subsoils.
